# Job Strain and Tobacco Smoking: An Individual-Participant Data Meta-Analysis of 166 130 Adults in 15 European Studies

**DOI:** 10.1371/journal.pone.0035463

**Published:** 2012-07-06

**Authors:** Katriina Heikkilä, Solja T. Nyberg, Eleonor I. Fransson, Lars Alfredsson, Dirk De Bacquer, Jakob B. Bjorner, Sébastien Bonenfant, Marianne Borritz, Hermann Burr, Els Clays, Annalisa Casini, Nico Dragano, Raimund Erbel, Goedele A. Geuskens, Marcel Goldberg, Wendela E. Hooftman, Irene L. Houtman, Matti Joensuu, Karl-Heinz Jöckel, France Kittel, Anders Knutsson, Markku Koskenvuo, Aki Koskinen, Anne Kouvonen, Constanze Leineweber, Thorsten Lunau, Ida E. H. Madsen, Linda L. Magnusson Hanson, Michael G. Marmot, Martin L. Nielsen, Maria Nordin, Jaana Pentti, Paula Salo, Reiner Rugulies, Andrew Steptoe, Johannes Siegrist, Sakari Suominen, Jussi Vahtera, Marianna Virtanen, Ari Väänänen, Peter Westerholm, Hugo Westerlund, Marie Zins, Töres Theorell, Mark Hamer, Jane E. Ferrie, Archana Singh-Manoux, G. David Batty, Mika Kivimäki, for the IPD-Work Consortium

**Affiliations:** 1 Finnish Institute of Occupational Health, Helsinki, Finland; 2 Institute of Environmental Medicine, Karolinska Institutet, Stockholm, Sweden; 3 School of Health Sciences, Jönköping University, Jönköping, Sweden; 4 Department of Public Health, Ghent University, Ghent, Belgium; 5 National Research Centre for the Working Environment, Copenhagen, Denmark; 6 Inserm U1018, Centre for Research in Epidemiology and Population Health, Villejuif, France; 7 Versailles-Saint Quentin University, Versailles, France; 8 Department of Occupational Medicine, Bispebjerg University Hospital, Copenhagen, Denmark; 9 Centre for Maritime Health and Safety, Esbjerg, Denmark; 10 School of Public Health, Université Libre de Bruxelles, Brussels, Belgium; 11 Institute for Medical Informatics, Biometry, and Epidemiology, University Duisburg-Essen, Essen, Germany; 12 Department of Cardiology, West-German Heart Center Essen, University Duisburg-Essen, Essen, Germany; 13 TNO, Hoofddorp, The Netherlands; 14 Department of Health Sciences, Mid Sweden University, Sundsvall, Sweden; 15 Department of Public Health, University of Helsinki, Helsinki, Finland; 16 Warsaw School of Social Sciences and Humanities, Wroclaw Faculty, Wroclaw, Poland; 17 Stress Research Institute, Stockholm University, Stockholm, Sweden; 18 Department of Epidemiology and Public Health, University College London, London, United Kingdom; 19 Department of Occupational and Environmental Medicine, Bispebjerg University Hospital, Copenhagen, Denmark; 20 Department of Public Health and Clinical Medicine, Occupational and Environmental Medicine, Umeå University, Umeå, Sweden; 21 Finnish Institute of Occupational Health, Turku, Finland; 22 Department of Public Health and Department of Psychology, University of Copenhagen, Copenhagen, Denmark; 23 Department of Medical Sociology, University of Düsseldorf, Düsseldorf, Germany; 24 Folkhälsan Research Center, Helsinki, Finland; 25 Department of Public Health, University of Turku, Turku, Finland; 26 Turku University Hospital, Turku, Finland; 27 Occupational and Environmental Medicine, Uppsala University, Uppsala, Sweden; 28 School of Community and Social Medicine, University of Bristol, Bristol, United Kingdom; Catholic University of Sacred Heart of Rome, Italy

## Abstract

**Background:**

Tobacco smoking is a major contributor to the public health burden and healthcare costs worldwide, but the determinants of smoking behaviours are poorly understood. We conducted a large individual-participant meta-analysis to examine the extent to which work-related stress, operationalised as job strain, is associated with tobacco smoking in working adults.

**Methodology and Principal Findings:**

*We analysed cross-sectional data from 15 European studies comprising 166 130 participants. Longitudinal data from six studies were used. Job strain and smoking were self-reported. Smoking was harmonised into three categories* never, ex- and current. We modelled the cross-sectional associations using logistic regression and the results pooled in random effects meta-analyses. Mixed effects logistic regression was used to examine longitudinal associations. Of the 166 130 participants, 17% reported job strain, 42% were never smokers, 33% ex-smokers and 25% current smokers. In the analyses of the cross-sectional data, current smokers had higher odds of job strain than never-smokers (age, sex and socioeconomic position-adjusted odds ratio: 1.11, 95% confidence interval: 1.03, 1.18). Current smokers with job strain smoked, on average, three cigarettes per week more than current smokers without job strain. In the analyses of longitudinal data (1 to 9 years of follow-up), there was no clear evidence for longitudinal associations between job strain and taking up or quitting smoking.

**Conclusions:**

Our findings show that smokers are slightly more likely than non-smokers to report work-related stress. In addition, smokers who reported work stress smoked, on average, slightly more cigarettes than stress-free smokers.

## Introduction

Tobacco smoking is an important risk factor for chronic diseases, most notably lung cancer and other pulmonary diseases, and as such a major contributor to the public health burden and healthcare costs worldwide [1], [2], [3], [4]. Understanding the determinants of tobacco smoking is important because it would help healthcare professionals, policy makers and individuals to develop and utilise smoking cessation strategies, thus reducing the disease burdern associated with the habit. To date, however, the determinants of smoking behaviour are not well understood.

Two possible important determinants of smoking are stress in general and work-related stress in particular. However, recent observational studies of the relationship between work stress and tobacco smoking have produced mixed findings, with positive, negative and null-associations reported [5], [6], [7], [8], [9]. In many studies the associations of tobacco smoking and work stress have differed by study population and gender [7], [10], [11]. Important limitations of previous studies are that few studies thus far have been sufficiently well powered to detect small or moderate associations or to investigate whether any associations differ in socio-demographic or other subgroups of participants.

In order to collate and add to the existing evidence, we have undertaken individual-participant meta-analyses of the associations of tobacco smoking with work-related stress. We used a large set of data pooled from 15 independent European studies with a common measure of work stress, operationalised as job strain [12], [13]. We also examined the associations of job strain and smoking in socio-demographic subgroups.

## Methods

### Ethical Approval

Each constituent study in the IPD-Work consortium was approved by the relevant local or national ethics committees and all participants gave informed consent to take part. Details of the ethical approval are provided in Appendix S1.

### Studies and Participants

We conducted individual-level meta-analyses using pooled data from 15 independent studies conducted between 1985 and 2008 in Belgium, Denmark, Finland, France, Germany, the Netherlands, Sweden and the UK. The “Individual-participant-data Meta-analysis of Working Populations” (IPD-Work) Consortium was established at the annual Four Centers Meeting in London, November 8, 2008. A pre-defined two-stage data acquisition protocol was used. The first stage involved the acquisition of baseline data on job strain as well as socio-demographic and lifestyle factors and the definition and harmonisation of these baseline characteristics across the studies. The second stage involves the acquisition of data on disease outcomes. Our meta-analyses were based on the first stage cross-sectional data and were thus conducted before any linkage to disease data. MOOSE checklist is provided in Appendix S2.

Details of the design and participants in the IPD-Work Consortium studies have been described previously [14] and are provided in Appendix S1. The following studies were included in our analyses: Belstress, Copenhagen Psychosocial Questionnaire version I (COPSOQ-I), Danish Work Environment Cohort Study (DWECS), Finnish Public Sector Study (FPS), Health and Social Support (HeSSup), Heinz Nixdorf Recall study (HNR), Intervention Project on Absence and Well-being (IPAW), Permanent Onderzoek Leefsituatie (POLS), Burnout, Motivation and Job Satisfaction study (Danish acronym PUMA), Swedish Longitudinal Occupational Survey of Health (SLOSH), Whitehall II and Work Lipids and Fibrinogen (WOLF) Norrland and Stockholm) Participants with complete data on job strain and smoking, as well as age, sex, and socioeconomic position were included in our analyses (Table 1).

**Table 1:** Summary of studies and participants.

Study^1^ (country)	N participants^2^	N (%) female	Age: mean (SD) range
Belstress (Belgium)	20 815	4 853 (23.3)	45.5 (5.9) 33–61
COPSOQ-I (Denmark)	1 768	857 (48.5)	40.7 (10.6) 20–60
DWECS (Denmark)	5 571	2 605 (46.8)	41.8 (11.0) 18–69
FPS (Finland)	44 696	36 153 (80.9)	44.5 (9.4) 17–64
Gazel (France)	11 354	3 136 (27.6)	50.3 (3.0) 43–58
HeSSup (Finland)	15 106	8 524 (56.4)	39.6 (10.3) 20–54
HNR (Germany)	1 827	747 (40.9)	53.4 (5.0) 45–73
IPAW (Denmark)	2 021	1 350 (66.8)	41.3 (10.5) 18–68
POLS (the Netherlands)	20 633	8 764 (42.5)	38.5 (11.3) 15–85
PUMA (Denmark)	1 844	1 521 (82.5)	42.6 (10.3) 18–69
SLOSH (Sweden)	10 887	5 875 (54.0)	47.7 (10.8) 19–68
Still Working (Finland)	9 065	2 070 (22.8)	40.9 (9.1) 18–65
Whitehall II (United Kingdom)	10 198	3 374 (33.1)	44.4 (6.0) 34–56
WOLF Norrland (Sweden)	4 698	779 (16.6)	44.1 (10.3) 19–65
WOLF Stockholm (Sweden)	5 647	2 442 (43.2)	41.5 (11.0) 19–70
**All**	166 130	83 050 (50.0)	43.8, 15–85

1 Study acronyms: COPSOQ-I: Copenhagen Psychosocial Questionnaire version I; DWECS: Danish Work Environment Cohort Study; FPS: Finnish Public Sector Study; HeSSup: Health and Social Support; HNR: Heinz Nixdorf Recall study; IPAW: Intervention Project on Absence and Well-being; POLS: Permanent Onderzoek Leefsituatie; PUMA: Burnout, Motivation and Job Satisfaction study; SLOSH: Swedish Longitudinal Occupational Survey of Health; WOLF: Work Lipids and Fibrinogen.

2 Participants with complete data on job strain, age, sex and socioeconomic position. SD: standard deviation.

We estimated study-specific cross-sectional associations of tobacco smoking and psychosocial job strain in 15 studies, based on data from 166 130 individuals (mean age: 43.8 years). In addition, the associations of smoking and work stress in socio-demographic subgroups were investigated in studies in which we had access to individual-level data (n = 134 293, Figure S1). Longitudinal associations of smoking and job strain were examined using individual-level repeated measurements data from six studies, in which these data were available (n = 52 024; Figure S1).

### Ascertainment of Tobacco Smoking and Work Stress

Tobacco smoking was ascertained from participant-completed questionnaires in all studies. Smokers were categorised into never, ex- and current smokers. Job strain was ascertained in all studies using questions from the Job Content Questionnaire (JCQ) and Demand-Control Questionnaire (DCQ) [12], [13]. A description of the questionnaires and job demand and job control scales is provided elsewhere [15]. Mean response score for job demands items and mean response score for job control items were calculated for each participant. Job strain was defined as having a high demand (>study-specific median score) and a low control score (<study-specific median score). All other combinations of job demands and job control, including the values equal to the median values, were assigned to the no strain-category. Participants with missing data on more than half of the job demands or job control items (n = 1 714, 1%) were excluded from the analyses.

### Covariates

Information on sex and age was obtained from population registries or baseline interview (in COPSOQ-I, DWECS, FPS, Gazel, HNR, IPAW, PUMA, SLOSH, Still Working, WOLF Norrland and WOLF Stockholm) or from participant-completed questionnaires (in Belstress, HeSSup, POLS and Whitehall II). Sex was modelled as binary and age as a continuous variable (years). Socioeconomic position was defined based on the occupational title obtained from employers’ or other registries (in COPSOQ-I, DWECS, FPS, Gazel, IPAW, PUMA, and Still Working) or participant-completed questionnaires (in Belstress, HeSSup, HNR, POLS, SLOSH, Whitehall II, WOLF Norrland and WOLF Stockholm). In HeSSup socioeconomic position was defined based on the participant’s self-reported highest educational qualification. Socioeconomic position was categorised in all studies as low (e.g. cleaners, maintenance workers), intermediate (e.g. registered nurses, technicians) or high (e.g. teachers, physicians). Participants who were self-employed or who had missing data on socioeconomic position were included in the analyses in the “other” socioeconomic position category (n = 2 173, 1.3%).

### Statistical Analyses

We used individual-level data provided by the investigators in Belstress, FPS, Gazel, HeSSup, HNR, Still Working, SLOSH, Whitehall II, WOLF Norrland and WOLF Stockholm studies. Investigators in COPSOQ-I, DWECS, IPAW, POLS and PUMA undertook the statistical analyses themselves according to our instructions and provided us with the study-specific results.

Individual-participant data meta-analyses can take a one-stage or two-stage approach. In the one-stage approach, individual-level data are pooled and analysed as clustered data, with study as the cluster; in the two-stage approach the effect estimates are calculated for each study separately and subsequently pooled using standard meta-analytical methods [16], [17], [18]. These approaches have been shown to provide similar results and the choice of approach depends on the research questions and available data.

Our main meta-analyses were done using the two-stage approach because we had access to individual-level data from 11 studies but only aggregate data from five studies (COPSOQ-I, DWECS, IPAW, POLS and PUMA). We used logistic regression models to estimate study-specific associations of smoking and job strain and pooled the resulting estimates and their standard errors using fixed effect and random effects meta-analyses [19]. We quantified heterogeneity in the pooled effect estimates using the I^2^ statistic, which indicates the proportion of the total variation in the estimates that is due to between-studies variation [20]. The one-stage approach was used to investigate exposure-covariate interactions and bidirectional longitudinal associations of smoking and job strain at baseline and follow-up, because this approach provides a flexible way of investigating individual-level interactions [18], [21], [22], [23]. Our one-stage analyses were conducted using mixed effects logistic regression with study as the random effect when the outcomes were rare, and using modified Poisson regression with robust standard errors and study as the cluster-variable when the outcomes were common [24]. Interactions were investigated by stratifying the mixed effects logistic models for sex, age group and socioeconomic position, and tested by including an interaction term (smoking*covariate) in the model that also contained the main effects.

All meta-analyses and statistical analyses in the pooled individual-level data were performed using Stata SE 11.0 (Stata Corporation, College Station, Texas, USA). In POLS the study-specific analyses were undertaken by the study team using SPSS 17 (SPSS Inc., Chicago, Illinois, USA) and in COPSOQ-I, DWECS, IPAW and PUMA using SAS 9 (SAS Institute Inc., Cary, North Carolina, USA).

## Results

### Tobacco Smoking and Job Strain

The characteristics of participants included in our analyses are shown in Table 1. Of the 166 130 participants, 26 415 (15.9%) reported job strain, 42 146 (25.4%) were current smokers, 54 029 (32.5%) ex-smokers and 69 955 (42.1%) never smokers.

**Figure 1:**
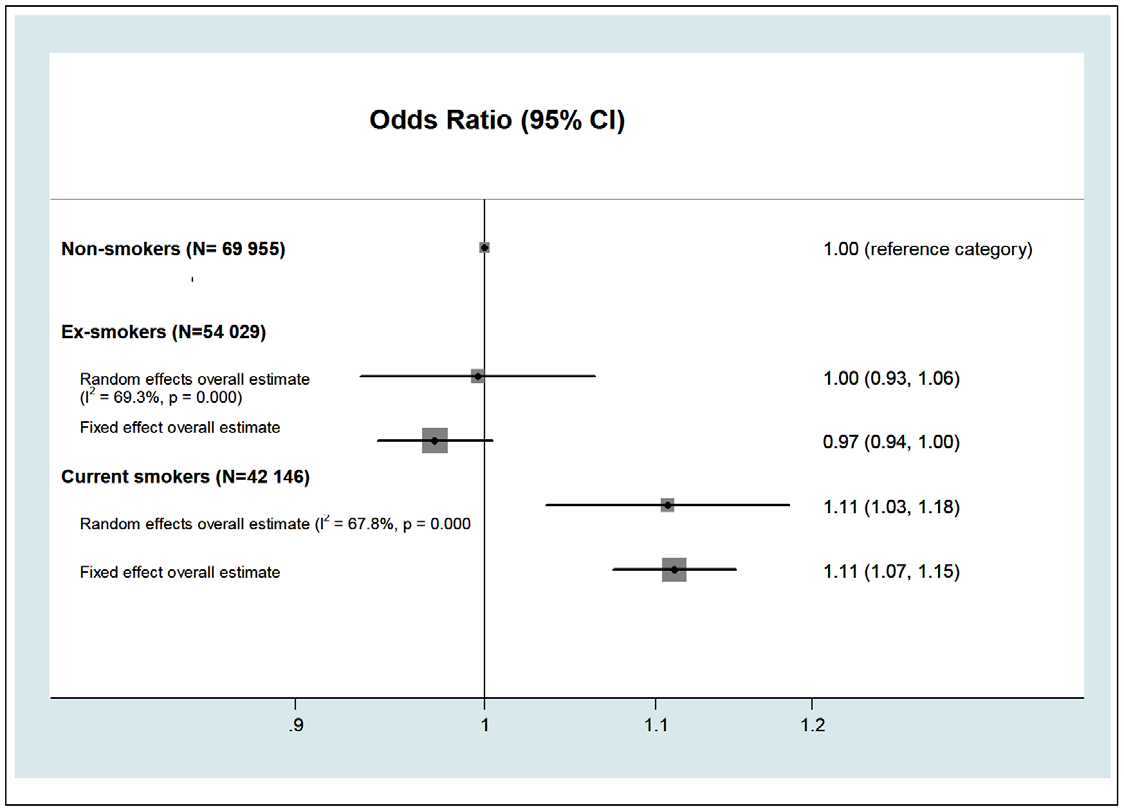
Association of tobacco smoking and job strain (adjusted for age, sex and socioeconomic status) (N  = 166 130).

A meta-analysis of smoking and job strain is shown in Figure 1. In the age, sex and socioeconomic position -adjusted analyses, compared to never-smokers, current smokers were, on average, 11% more likely to experience job strain (pooled random effects odds ratio (OR): 1.11, 95% confidence interval (CI): 1.03, 1.18). The odds of reporting job strain were similar among ex-smokers and never-smokers after the same adjustments (OR: 1.00, 95% CI: 0.93, 1.06). There was heterogeneity in the study-specific effect estimates comparing ex-smokers to never-smokers (I^2^: 69.3%) and current smokers to never-smokers (I^2^: 67.8%). The study-specific effect estimates are shown in Figure S2.

Current smokers in our study population smoked, on average, 102 (standard deviation: 63) cigarettes in a typical week. Among current smokers, the difference in the mean number of cigarettes smoked during an average week between individuals who reported job strain and those who did not are shown in Table 2. Current smokers who reported job strain smoked, on average, three cigarettes more per week than current smokers who did not report job strain.

**Table 2:** Difference in mean number of cigarettes in an average week between current smokers with and without job strain.

Job strain status (n = 25 561)	Mean (SD) number of cigarettes	Difference in means (95% CI)	
		Random effects meta-analysis	Fixed effect meta-analysis
No job strain (n = 20 652)	99.9 (60.7)	1 (reference category)	1 (reference category)
Job strain (n = 4 909)	103.3 (61.2)	2.96 (0.87, 5.04)	2.72 (1.04, 4.41)
		I2 = 19.4%, p = 0.270	

NB: Analyses include participants who were current smokers at baseline, for whom we had access to individual-level data and who had smoking intensity data available and were adjusted for age, sex and socioeconomic position.

We investigated the availability of individual-level data in the IPD-Work Consortium as a possible source of heterogeneity by stratifying our meta-analyses by the availability of individual-level data (Figure S3). The pooled effect estimates from the studies in which we had no access to individual-level data were more extreme yet less precisely estimated than those from the studies which had these data available.

### Stratified Analyses in Socio-demographic Subgroups

The findings from the meta-analysis stratified by demographic covariates (sex, age and socioeconomic position) and based on individual-level data are shown in Table S1. The associations between smoking and job strain were similar in men and women and any differences in these associations between age groups and socioeconomic positions were small.

### Longitudinal Analyses of Smoking and Work Stress

Longitudinal analyses were based on pooled repeated measurements data from Belstress, FPS, HeSSup, SLOSH, Whitehall II and WOLF Norrland and the duration of follow-up varied between 1 and 9 years. The associations between job strain at baseline and taking up smoking by follow-up (among baseline never- and ex-smokers grouped together) are presented in Table 3. The same associations are presented separately for baseline never-and ex-smokers in Table S2. We found no clear evidence for an association between either job strain at baseline, or change in job strain status between baseline and follow-up, and taking up smoking. However, all these associations were imprecisely estimated. The associations between job strain at baseline and quitting smoking by follow-up (among baseline smokers) are shown in Table 4. Job strain at baseline was associated with neither taking up nor quitting smoking by follow-up. The associations between smoking at baseline and job strain at follow-up, stratified by baseline job strain, are shown in Table S3. There was no clear evidence for smoking at baseline being associated with changing from no strain to strain or vice versa between baseline and follow-up.

**Table 3:** Longitudinal associations between job strain and taking up smoking among baseline never- and ex-smokers (n = 42 049)^1^.

Job strain at baseline	N participants	N (%) taking up smoking	OR (95% CI)^2^ for being smoker at follow-up
No	35 649	947 (2.7)	1 (reference category)
Yes	6 409	186 (2.9)	1.03 (0.87, 1.21)
Job strain at baseline and follow-up			
No and no	31 968	854 (2.7)	1 (reference category)
No and yes	3 681	93 (2.5)	0.91 (0.73, 1.14)
Yes and no	3 793	104 (2.7)	0.94 (0.76, 1.16)
Yes and yes	2 607	82 (3.1)	1.13 (0.89, 1.43)

1 Studies and follow-up times: Belstress (4–7 years), FPS (2–4 years), HeSSup (5 years), SLOSH (1–4 years), WOLF Norrland (3–7 years) and Whitehall II (3–9 years.).

2 Effect estimates from a mixed effects logistic model, adjusted for baseline age, sex and baseline socioeconomic position, with study as the random effect.

**Table 4:** Longitudinal associations between job strain at baseline and quitting smoking among baseline smokers (n = 9 975)^1^.

Job strain at baseline	N participants	N (%) quitting smoking	OR (95% CI)^2^ for quitting smoking
No	8 149	1 574 (19.3)	1 (reference category)
Yes	1 826	298 (16.3)	0.91 (0.79, 1.04)
Job strain at baseline and follow-up			
No and no	7 192	1 411 (19.6)	1 (reference category)
No and yes	957	163 (17.0)	0.86 (0.71, 1.03)
Yes and no	1 029	167 (16.3)	0.85 (0.71, 1.02)
Yes and yes	797	131 (16.4)	0.95 (0.77, 1.16)

1 Studies and follow-up times: Belstress (4–7 years), FPS (2–4 years), HeSSup (5 years), SLOSH (1–4 years), WOLF Norrland (3–7 years) and Whitehall II (3–9 years.).

2 Effect estimates from a mixed effects logistic model, adjusted for baseline age, sex and baseline socioeconomic position, with study as the random effect.

## Discussion

In our pooled analyses of data drawn from 15 European studies, current smokers had approximately 11% higher odds of reporting job strain than never smokers. However, no difference in job strain was observed between ex-smokers and never-smokers. Job strain was associated with smoking dose in current smokers: those who reported job strain smoked, on average, three cigarettes more in an average week than current smokers who did not report job strain. No difference in job strain was observed between ex-smokers and never-smokers. Furthermore, we observed no clear evidence for longitudinal associations between smoking and job strain during follow-ups varying between 1 and 9 years.

Given that the excess of smokers in employees with job strain was relatively small, insufficient statistical power to detect modest associations is a potential contributing factor to inconsistencies in prior evidence [5], [6], [7], [8], [9]. The present analyses, however, were based on over three times as large a sample size as, to our knowledge, the largest previous study on this topic [25]. Our findings replicate the higher smoking intensity among stressed employees observed in that study [25] as well as the association between job strain and current smoking observed in other studies [5], [6], [7], [8], [9].

The direction of the association between job strain and smoking is not clear. Our cross-sectional findings indicate that they co-occur. We hypothesised that job strain could lead to taking up or being unable to quit smoking [10]; it is also possible that as a part of an unhealthy lifestyle smoking could lead to job strain. We investigated the direction of the relationship between smoking and job strain using repeat measures of both at baseline and follow-up in a subset of six studies (Belstress, Finnish Public Sector, HeSSup, SLOSH, WOLF Norrland and Whitehall II). However, our longitudinal analyses provided no clear evidence of a temporal association between smoking status and job strain, supporting the findings from the cross-sectional meta-analyses that smoking and work stress co-occur, but suggesting the two are unlikely to be causally related. It is, however, possible that the null findings in some of the longitudinal analyses reflect low statistical power to detect modest associations. Reporting job strain at baseline as well as follow-up was not associated with taking up smoking among baseline never- and ex-smokers grouped together (Table 3: OR: 1.03, 95% CI: 0.87, 1.21). When analysed separately, the majority of those who took up smoking during the study follow-up were baseline ex-smokers, with fewer than 1% of the never smokers starting to smoke (Table S2). We observed no association between job strain at baseline and taking up smoking by follow-up among baseline ex-smokers but, due to small numbers, particularly the analyses among baseline never smokers had insufficient power to allow reliable interpretation of the findings. The complementary analysis among baseline smokers suggests that smokers with job strain at baseline were 9% less likely to give up smoking than smokers without job strain (Table 4: OR: 0.91, 95% CI: 0.79, 1.04) - an effect size consistent with the cross-sectional analysis based on 166 000 participants.

Important strengths of our investigation were that our analyses were based on published and unpublished individual-level data from a large number of participants. Such individual-data meta-analysis is a strong study design, which reduces the possibility of publication bias that can hamper evidence from single studies and literature-based meta-analyses [26]. Work stress was defined in all studies using a widely accepted, harmonised measure, job strain [12]. The job strain measure is based on self-reported subjective assessment of attributes of the job, job demand and control and thus the possibility of reporting bias cannot be excluded. However, the effects of work-related stress assessed by self-report match well with those based on objective indicators [27]. Smoking was ascertained from participants’ self-report in all the studies and there is evidence that self-report is a fairly accurate measure of smoking behaviour [28], with demonstrated predictive validity [29]. However, due to the way questions on smoking were asked in some studies, it is possible that some ex-smokers who had given up smoking a considerable time before the study baseline have been misclassified as never smokers. Misclassification of the participant-reported measures may have lead to an under- or over-estimate of the study-specific effect estimates and, as a result, introduced heterogeneity in our meta-analyses. It is also possible that some of our findings have been influenced by residual confounding from unmeasured confounders, such as mood disorders, addiction or personality type [30], [31], [32], [33], or long working hours or overtime work [34]. Future research would help to understand the role of these factors in the association between smoking habits and job strain.

### Conclusions

Our findings, based on individual-level data from 15 European studies, show that current smokers are more likely than non-smokers to report job strain. Work-related psychosocial stress was also associated with smoking intensity, with smokers who reported job strain smoking, on average, three cigarettes per week more than smokers not reporting job strain. These findings, which identify job strain as a factor that co-occurs with smoking, could be helpful in designing and implementing smoking cessation initiatives. Smokers who report job strain could be identified as being in slightly higher risk of smoking-associated disease due to their higher smoking intensity and in need of particular support in cutting down and quitting smoking.

## Supporting Information

Figure S1 **Studies and participants included in the analyses.**
(DOC)

Figure S2 **Associations of tobacco smoking and job strain (adjusted for age, sex and socioeconomic position).**
(DOC)

Figure S3 **Associations of smoking and job strain, stratified by the availability of individual-level data (adjusted for age, sex and socioeconomic position).**
(DOC)

Table S1 **Associations of smoking and work stress in demographic subgroups^1^.**
(DOC)

Table S2 **Longitudinal associations between job strain and taking up smoking among baseline never- and ex-smokers.**
(DOC)

Table S3 **Longitudinal associations between smoking at baseline and job strain at follow-up, stratified by baseline job strain category^1^.**
(DOC)

Appendix S1 **Study design and recruitment of participants.**
(DOC)

Appendix S2 **MOOSE checklist.**
(DOC)
