# Supplementary material for: Job Strain and Tobacco Smoking: An Individual-Participant Data Meta-Analysis of 166 130 Adults in 15 European Studies
Source: PLoS One. 2012 Jul 6;7(7):e35463. doi: 10.1371/journal.pone.0035463 (PMC3391192; doi:10.1371/journal.pone.0035463)
Supplement: Figure S1 — Studies and participants included in the analyses. (DOC) [file pone.0035463.s001.doc]

**Figure S1. Studies and participants included in the analyses**

**IPD Work meta-analysis:**

15 studies: Belstress, COPSOQ-I, DWECS, FPS, Gazel, HeSSup, HNR, IPAW, POLS, PUMA, SLOSH, Still Working, Whitehall II WOLF Norrland and WOLF Stockholm.

**Individual-level pooled data with repeated measurements** (repeated measures analyses of tobacco smoking and work stress):

5 studies: Belstress, FPS, HeSSup, SLOSH, WOLF Norrland and Whitehall II. N=52 024.

**Individual-level pooled data**

**(analyses of tobacco smoking and work stress in subgroups):**

10 studies: Beltress, FPS, Gazel, HeSSup, HNR, SLOSH, Still Working, Whitehall II, WOLF Norrland and WOLF Stockholm.

N=134 293

**Meta-analyses of tobacco smoking and work stress:**

15 studies: Belstress, COPSOQ-I, DWECS, FPS, Gazel, HeSSup, HNR, IPAW, POLS, PUMA, SLOSH, Still Working, Whitehall II WOLF Norrland and WOLF Stockholm. N=166 130.
