# Supplementary material for: Job Strain and Tobacco Smoking: An Individual-Participant Data Meta-Analysis of 166 130 Adults in 15 European Studies
Source: PLoS One. 2012 Jul 6;7(7):e35463. doi: 10.1371/journal.pone.0035463 (PMC3391192; doi:10.1371/journal.pone.0035463)
Supplement: Figure S2 — Associations of tobacco smoking and job strain (adjusted for age, sex and socioeconomic position). (DOC) [file pone.0035463.s002.doc]

**Figure S2. Associations of tobacco smoking and job strain (adjusted for age, sex and socioeconomic position)**

NOTE: Weights are from random effects analysis

.

.

.

**Reference category:**

**Non-smokers**

(N= 69 955)

Ex-smokers

Belstress

COPSOQ

DWECS

FPS

Gazel

HNR

HeSSup

IPAW

POLS

PUMA

SLOSH

Still Working

WOLF Norrland

WOLF Stockholm

Whitehall

Random effects overall estimate (I^2^= 69.5%, p = 0.000)

Fixed effect overall estimate

Current smokers

Belstress

COPSOQ

DWECS

FPS

Gazel

HNR

HeSSup

IPAW

POLS

PUMA

SLOSH

Still Working

WOLF Norrland

WOLF Stockholm

Whitehall

Random effects overall estimate (I^2^= 67.9%, p = 0.000)

Fixed effect overall estimate

Study

6623

432

1283

17276

4417

672

4479

466

5958

454

2803

2887

1440

1568

3269

6749

682

2226

7951

2022

554

3962

601

7806

668

1743

2992

887

1424

1879

smokers

Number of

31.82

24.43

23.03

38.65

38.90

36.78

29.65

23.06

28.88

24.66

25.75

31.85

30.65

27.77

32.06

32.42

38.57

39.96

17.79

17.81

30.32

26.23

29.74

37.83

36.28

16.01

33.01

18.88

25.22

18.43

smokers

%

1.02 (0.93, 1.12)

1.09 (0.83, 1.43)

1.15 (0.99, 1.34)

0.89 (0.84, 0.95)

1.00 (0.88, 1.13)

0.66 (0.46, 0.96)

0.84 (0.76, 0.94)

1.22 (0.92, 1.62)

1.13 (1.02, 1.25)

0.82 (0.61, 1.10)

1.07 (0.95, 1.20)

1.00 (0.86, 1.15)

1.18 (0.97, 1.45)

1.01 (0.85, 1.21)

0.90 (0.79, 1.03)

1.00 (0.93, 1.06)

0.97 (0.94, 1.00)

1.23 (1.12, 1.34)

1.20 (0.89, 1.63)

1.18 (0.99, 1.40)

0.98 (0.91, 1.05)

1.28 (1.10, 1.48)

1.06 (0.76, 1.50)

1.05 (0.94, 1.16)

1.09 (0.78, 1.53)

1.25 (1.14, 1.36)

0.81 (0.58, 1.14)

1.31 (1.15, 1.49)

0.98 (0.85, 1.13)

0.93 (0.73, 1.18)

1.03 (0.86, 1.23)

1.09 (0.94, 1.26)

1.11 (1.03, 1.18)

1.11 (1.07, 1.15)

OR (95% CI)

9.24

3.77

7.07

10.35

7.90

2.46

8.70

3.55

8.84

3.35

8.26

7.26

5.39

6.17

7.68

100.00

9.83

3.47

6.58

10.33

7.54

2.94

9.17

2.97

9.69

2.95

8.18

7.68

4.69

6.49

7.48

100.00

(D+L)

Weight

%

1.02 (0.93, 1.12)

1.09 (0.83, 1.43)

1.15 (0.99, 1.34)

0.89 (0.84, 0.95)

1.00 (0.88, 1.13)

0.66 (0.46, 0.96)

0.84 (0.76, 0.94)

1.22 (0.92, 1.62)

1.13 (1.02, 1.25)

0.82 (0.61, 1.10)

1.07 (0.95, 1.20)

1.00 (0.86, 1.15)

1.18 (0.97, 1.45)

1.01 (0.85, 1.21)

0.90 (0.79, 1.03)

1.00 (0.93, 1.06)

0.97 (0.94, 1.00)

1.23 (1.12, 1.34)

1.20 (0.89, 1.63)

1.18 (0.99, 1.40)

0.98 (0.91, 1.05)

1.28 (1.10, 1.48)

1.06 (0.76, 1.50)

1.05 (0.94, 1.16)

1.09 (0.78, 1.53)

1.25 (1.14, 1.36)

0.81 (0.58, 1.14)

1.31 (1.15, 1.49)

0.98 (0.85, 1.13)

0.93 (0.73, 1.18)

1.03 (0.86, 1.23)

1.09 (0.94, 1.26)

1.11 (1.03, 1.18)

1.11 (1.07, 1.15)

9.24

3.77

7.07

10.35

7.90

2.46

8.70

3.55

8.84

3.35

8.26

7.26

5.39

6.17

7.68

100.00

9.83

3.47

6.58

10.33

7.54

2.94

9.17

2.97

9.69

2.95

8.18

7.68

4.69

6.49

7.48

100.00

(D+L)

Weight

%

1

.46

1

2.17
