# Supplementary material for: Job Strain and Tobacco Smoking: An Individual-Participant Data Meta-Analysis of 166 130 Adults in 15 European Studies
Source: PLoS One. 2012 Jul 6;7(7):e35463. doi: 10.1371/journal.pone.0035463 (PMC3391192; doi:10.1371/journal.pone.0035463)
Supplement: Figure S3 — Associations of smoking and job strain, stratified by the availability of individual-level data (adjusted for age, sex and socioeconomic position). (DOC) [file pone.0035463.s003.doc]

**Figure S3. Associations of smoking and job strain, stratified by the availability of individual-level data (adjusted for age, sex and socioeconomic position)**

.

.

.

.

**Ex-smokers, no individual-level data**

Random effects (I-squared = 16.6%, p = 0.309)

Fixed effect

Ex-smokers, with individual-level data

Random effects (I-squared = 65.2%, p = 0.002)

Fixed effect

**Current smokers, no individual-level data**

Random effects (I-squared = 33.7%, p = 0.197)

Fixed effect

**Current smokers, with individual-level data**

Random effects (I-squared = 72.2%, p = 0.000)

Fixed effect

1.11 (1.01, 1.21)

1.11 (1.03, 1.20)

0.96 (0.90, 1.03)

0.94 (0.91, 0.98)

1.16 (1.03, 1.30)

1.20 (1.11, 1.29)

1.09 (1.01, 1.19)

1.09 (1.05, 1.13)

Odds ratio (95% Confidence Interval)

1

.769

1

1.3
