# Supplementary material for: Job Strain and Tobacco Smoking: An Individual-Participant Data Meta-Analysis of 166 130 Adults in 15 European Studies
Source: PLoS One. 2012 Jul 6;7(7):e35463. doi: 10.1371/journal.pone.0035463 (PMC3391192; doi:10.1371/journal.pone.0035463)
Supplement: Table S1 — Associations of smoking and work stress in demographic subgroups1. (DOC) [file pone.0035463.s004.doc]

**Table S1. Associations of smoking and work stress in demographic subgroups1**

|  | **N (n with job strain)** **% with job strain** | **OR (95% CI) for job strain ^2^** |
| --- | --- | --- |
| **Ex-smokers (n=45 434) vs. non-smokers (n=58 702)** | | |
| All | 45 434 (6 876) 15.1% | 0.93 (0.90, 0.97) |
| Stratified by sex |  |  |
| Men | 24 246 (3 008) 12.4 % | 0.95 (0.90, 1.00) |
| Women | 21 188 (3 868) 18.3 % | 0.96 (0.92, 1.00) |
| p for interaction ^3^ |  | 0.019 |
| Stratified by age |  |  |
| <50 years | 27 610 (4 226) 15.3 % | 0.96 (0.92, 1.00) |
| >=50 years | 17 824 (2 656) 14.9 % | 0.91 (0.86, 0.97) |
| p for interaction ^3^ |  | 0.1 |
| Stratified by socioeconomic position | |  |
| Low | 12 184 (2 507) 20.6 % | 0.95 (0.90, 1.02) |
| Intermediate | 21 682 (3 661) 16.9 % | 0.95 (0.90, 0.99) |
| High | 11 022 (645) 5.9 % | 0.83 (0.75, 0.91) |
| p for interaction ^3^ |  | 0.4 |
| **Current smokers (n=30 163) vs. non-smokers (n=58 702)** | | |
| All | 30 163 (5 617) 18.6% | 1.07 (1.03, 1.11) |
| Stratified by sex |  |  |
| Men | 16 915 (2 677) 15.8 % | 1.13 (1.07, 1.20) |
| Women | 13 248 (2 940) 22.2 % | 1.06 (1.00, 1.11) |
| p for interaction ^3^ |  | 0.7 |
| Stratified by age |  |  |
| <50 years | 21 138 (4 008) 19.0 % | 1.11 (1.06, 1.16) |
| >=50 years | 9 025(1 609) 17.8 % | 1.03 (0.96, 1.10) |
| p for interaction ^3^ |  | 0.018 |
| Stratified by socioeconomic position | |  |
| Low | 11 590 (2 581) 22.3 % | 1.06 (1.00, 1.13) |
| Intermediate | 13 778 (2 636) 19.1 % | 1.08 (1.02, 1.14) |
| High | 4 351 (327) 7.5 % | 1.09 (0.96, 1.24) |
| p for interaction ^3^ |  | 0.034 |

^1^ In pooled set of individual-level data from ten studies: Belstress, FPS, Gazel, HeSSup, HNR, SLOSH, Still Working, Whitehall II, WOLF Norrland and WOLF Stockholm (N=134 293).

^2^ Odds ratios from a mixed effects logistic model with job strain as the outcome, smoking category as the main exposure, age, sex and socioeconomic position as covariates (where appropriate) and study as the random effect.

^3^ p-value for smoking*covariate
