# Supplementary material for: Job Strain and Tobacco Smoking: An Individual-Participant Data Meta-Analysis of 166 130 Adults in 15 European Studies
Source: PLoS One. 2012 Jul 6;7(7):e35463. doi: 10.1371/journal.pone.0035463 (PMC3391192; doi:10.1371/journal.pone.0035463)
Supplement: Table S2 — Longitudinal associations between job strain and taking up smoking among baseline never- and ex-smokers. (DOC) [file pone.0035463.s005.doc]

**Table S2. Longitudinal associations between job strain and taking up smoking among baseline never- and ex-smokers**1

|  | **N participants** | **N (%) taking up smoking** | **OR (95% CI)^2^ for being smoker at follow-up** |
| --- | --- | --- | --- |
| **Never smokers (n=22 936)** |  |  |  |
| Job strain at baseline |  |  |  |
| No | 19 412 | 99 (0.5) | 1 (reference category) |
| Yes | 3 524 | 25 (0.7) | 1.38 (0.87, 2.21) |
| Job strain at baseline and follow-up |  |  |  |
| No and no | 17 357 | 93 (0.5) | 1 (reference category) |
| No and yes | 2 055 | 6 (0.3) | 0.48 (0.20, 1.13) |
| Yes and no | 2 060 | 15 (0.7) | 1.27 (0.71, 2.27) |
| Yes and yes | 1 464 | 10 (0.7) | 1.32 (0.67, 2.62) |
| **Ex-smokers (n=19 113)** |  |  |  |
| Job strain at baseline |  |  |  |
| No | 16 237 | 848 (5.2) | 1 (reference category) |
| Yes | 2 876 | 161 (5.6) | 1.01 (0.84, 1.21) |
| Job strain at baseline and follow-up |  |  |  |
| No and no | 14 611 | 761 (5.2) | 1 (reference category) |
| No and yes | 1 626 | 87 (5.3) | 1.01 (0.80, 1.28) |
| Yes and no | 1 733 | 89 (5.1) | 0.90 (0.72, 1.14) |
| Yes and yes | 1 143 | 72 (6.3) | 1.18 (0.92, 1.53) |

^1^ Studies and follow-up times: Belstress (4-7 years), FPS (2-4 years), HeSSup (5 years), SLOSH (1-4 years), WOLF Norrland (3-7 years) and Whitehall II (3-9 years.)

^2^ Effect estimates from a mixed effects logistic model, adjusted for baseline age, sex and baseline socioeconomic position, with study as the random effect.
