# Supplementary material for: Job Strain and Tobacco Smoking: An Individual-Participant Data Meta-Analysis of 166 130 Adults in 15 European Studies
Source: PLoS One. 2012 Jul 6;7(7):e35463. doi: 10.1371/journal.pone.0035463 (PMC3391192; doi:10.1371/journal.pone.0035463)
Supplement: Table S3 — Longitudinal associations between smoking at baseline and job strain at follow-up, stratified by baseline job strain category1. (DOC) [file pone.0035463.s006.doc]

**Table S3. Longitudinal associations between smoking at baseline and job strain at follow-up, stratified by baseline job strain category 1**

| **Baseline exposure** | **N participants** | **N (%) developing job strain** | **OR (95% CI)^2^ for job strain at follow-up** |
| --- | --- | --- | --- |
| No job strain at baseline (n=43 798) |  |  |  |
| Non-smoker | 19 412 | 2 055 (10.6) | 1 |
| Ex-smoker | 16 237 | 1 626 (10.0) | 0.94 (0.88, 1.01) |
| Current smoker | 8 149 | 957 (11.7) | 1.03 (0.94, 1.12) |
| Job strain at baseline (n=8 226) |  | **N (%) no job strain at follow-up** | **IRR (95% CI)^3^ for no job strain at follow-up** |
| Non-smoker | 3 524 | 2 060 (58.5) | 1 |
| Ex-smoker | 2 576 | 1 733 (60.3) | 1.05 (1.02, 1.07) |
| Current smoker | 1 826 | 1 029 (56.4) | 0.99 (0.93, 1.05) |

^1^ Studies and follow-up times: Belstress (4-7 years), FPS (2-4 years), HeSSup (5 years), SLOSH (1-4 years) WOLF Norrland (3-7 years) and Whitehall II (3-9 years.) N= 52 024.

^2^ Effect estimates from a mixed effects logistic model, adjusted for baseline age, sex and baseline socioeconomic position, with study as the random effect.

^3^ Effect estimates from a modified Poisson model, adjusted for baseline age, sex and baseline socioeconomic position, with robust standard errors and study as the cluster variable.

IRR: incidence rate ratio
