# Supplementary material for: Job Strain and Tobacco Smoking: An Individual-Participant Data Meta-Analysis of 166 130 Adults in 15 European Studies
Source: PLoS One. 2012 Jul 6;7(7):e35463. doi: 10.1371/journal.pone.0035463 (PMC3391192; doi:10.1371/journal.pone.0035463)
Supplement: Appendix S1 — Study design and recruitment of participants. (DOC) [file pone.0035463.s007.doc]

**Web Appendix 1. Study design and recruitment of participants**

*Belstress*

Belstress is a prospective cohort study set up to investigate the associations between work-related stress and health outcomes. Between 1994 and 1998, 21 419 people aged 35-59 were recruited into the study from the payroll records of 25 large companies or public administrations [1, 2]. Of these, 21 024 men and women had data on job strain and were eligible for our meta-analyses. The ethics committees of the University Hospital of Ghent and the Faculty of Medicine of the Université Libre de Bruxelles approved the Belstress study.

*Copenhagen Psychosocial Questionnaire version I (COPSOQ-I)*

The COPSOQ-I is a prospective cohort study of a random sample of Danish residents selected from the Danish population register. The participants were aged 20-60 years of age and were in paid employment at the study baseline in 1997. A baseline questionnaire and an invitation to take part was posted to 4 000 people and 2 454 individuals agreed to participate [3]. Of the 1 858 employed participants, 1 776 men and women had data on job strain and were eligible for our meta-analyses. In Denmark, questionnaire- and register-based studies do not require ethics committee approval. COPSOQ-I was approved by and registered with the Danish Data protection agency (registration number: 2008 - 54 - 0553).

*Danish Work Environment Cohort Study (DWECS)*

DWECS is a split panel survey of working age Danish people. The cohort was established in 1990, when a simple random sample of men and women, aged 18-59, was drawn from the Danish population register. The participants have been followed up at five year intervals and data from the year 2000 was used for the IPD-Work. That year 11 437 individuals were invited to participate and 8 583 agreed to do so [4, 5]. Of the 5 606 individuals who were employed, 5 574 had data on job strain and were eligible for our meta-analyses. In Denmark, questionnaire- and register-based studies do not require ethics committee approval. DWECS was approved by and registered with the Danish Data protection agency (registration number: 2007-54-0059).

*Finnish Public Sector study (FPS)*

The Finnish Public Sector study is a prospective cohort study comprising the entire public sector personnel of 10 towns (municipalities) and 21 hospitals in the same geographical areas. Participants, who were recruited from employers' records in 2000-2002, were individuals who had been employed in the study organisations for at least six months prior to data collection [6]. 48 592 individuals (9 337 men and 39 255 women aged 17 to 65) responded to the questionnaire. Of these, 48 034 had data on job strain and were eligible for our meta-analyses. Ethical approval was obtained from the ethics committee of the Finnish Institute of Occupational Health.

*Gazel*

Gazel is a prospective cohort study of 20 625 employees (15 011 men and 5 614 women) of France's national gas and electricity company, Electricité de France-Gaz de France (EDF-GDF) [7, 8] . Since the study baseline in 1989, when the participants were aged 35–50 years, they have been posted an annual follow-up questionnaire to collect data on health, lifestyle, individual, familial, social, and occupational factors. Job strain was measured in Gazel in 1997, which we treated as a baseline year for our analyses. The 11 448 individuals who participated at that time and had data on job strain were eligible for our meta-analysis. The GAZEL study received approval from the national commission overseeing ethical data collection in France (Commission Nationale Informatique et Liberté).

*Health and Social Support (HeSSup)*

The Health and Social Support (HeSSup) study is a prospective cohort study of a stratified random sample of the Finnish population in the following four age groups: 20–24, 30–34, 40–44, and 50–54. The participants were identified from the Finnish population register and posted an invitation to participate, along with a baseline questionnaire, in 1998. A follow-up questionnaire was sent to all participants still living in Finland in 2003 [9]. Job strain was measured in 1998 and of the 19 629 individuals who responded to the follow-up questionnaire, 17 102 had data on job strain and were eligible for our meta-analyses. The Turku University Central Hospital Ethics Committee approved the study.

*Heinz Nixdorf Recall study (HNR)*

The Heinz Nixdorf Recall Study is a prospective population-based cohort study of individuals randomly selected from the mandatory lists of residence in the metropolitan Ruhr area in Germany. Details of the study methods have been described previously [10, 11] . Briefly, 4 814 participants aged 45-75 years were enrolled at study baseline in 2000-2003. Job stress measures and comprehensive medical data were collected during the baseline examination. For the present analyses baseline job strain measures were available for 1 841 employed men and women. The HNR was approved by the institutional local ethical committees and a quality management system according to European industrial norms (DIN EN ISO 9001:2000) was applied

*Intervention Project on Absence and Well-being (IPAW)*

IPAW is a 5-year psychosocial work environment intervention study including 22 intervention and 30 control work places in three organisations (a large pharmaceutical company, municipal technical services and municipal nursing homes) in Copenhagen, Denmark [12, 13]. The baseline questionnaire was posted to all the employees at the selected work-sites between 1996 and 1997. Of the 2 721 employees who worked at the 52 IPAW sites, 2 068 men and women completed the baseline questionnaire. Psychological, social support and other interventions took place at 22 workplaces during 1996-98 at the organisational and interpersonal level. Job strain was measured in 1996-1997 and the 2 031 participants, who had data on job strain, were eligible for our meta-analysis. IPAW was approved by and registered with the Danish Data Protection Agency (registration number: 2000-54-0066).

*Permanent Onderzoek Leefsituatie (POLS)*

Permanent Onderzoek Leefsituatie (POLS) is a series of annual cross-sectional health and lifestyle surveys of Dutch men and women[14]. The participants are a representative sample of the Dutch population, drawn form the Municipal Population Register (Gemeentelijke Basis Administratie, GBA). Only those living in a private household were included. Most of the data collection is done using computer assisted personal interviewing. At study baseline in 1997- 2002, 59 441 men and women participated in the surveys. Of these, 24 761 were in paid employment, aged 15-85 and had job strain measure available and were eligible for our meta-analyses. POLS was approved by the medical ethics committee of the Netherlands Organisation for Applied Scientific Research.

*Burnout, Motivation and Job Satisfaction study (Danish acronym: PUMA)*

Burnout, Motivation and Job Satisfaction study (Danish acronym: PUMA) is an intervention study of burn-out among employees in the human service sector [15]. Selection criteria for the participating organisations was that they had between 200 and 500 employees, that occupational groups within each organisation were willing to participate and that the organisations would commit to the entire five-year study period. Participants gave consent to having their national identity numbers collected and used in later record linkages to Danish hospitalisation and cause of death registries (Hospitalsindlæggelsesregisteret, Dødsårsagsregisteret. At study baseline in 1999-2000, 1 914 participants agreed to take part. Of these, 1 847 individuals had data on job strain and were eligible for our meta-analyses. PUMA was approved by the Scientific Ethical Committees (Videnskabsetisk Komiteer) in the counties in which the study was conducted and approved by and registered with the Danish Data Protection Agency (registration number: 2000-54-0048).

*Swedish Longitudinal Occupational Survey of Health (SLOSH)*

Swedish Longitudinal Occupational Survey of Health (SLOSH) is an on-going prospective cohort study following up individuals who participated in the Swedish Work Environment Survey (SWES) in 2003 or 2005. SWES, conducted biennially by Statistics Sweden, is based on a sample of gainfully employed people aged 16-64 years drawn from the Labour Force Survey (LFS). These individuals were first sampled into LFS through stratification by county, sex, citizenship and inferred employment status.

Data from the 2006 and 2008 data collection waves of SLOSH were used in the IPD-Work analyses. In both years, data were collected using postal self-completion questionnaires. In 2006, 5 985 individuals responded to the questionnaire. Of these, 5 104 people worked at least 30% of full time working hours and had data on job strain and were thus eligible for our meta-analyses [16]. In 2008, a further 6 751 individuals responded to the questionnaire [17]. Of these, 5 895 men and women worked at least 30% of full time working hours and had data on job strain and were thus eligible for our meta-analyses. SLOSH has been approved by the Regional Research Ethics Board in Stockholm.

*Still Working*

Still Working is an ongoing prospective cohort study. In 1986, the employees (7 174 men and 2 108 women aged 18 to 65) at all Finnish centres of operation of Enso Gutzeit (a forestry products manufacturer) were invited to participate in a questionnaire survey on demographic, psychosocial and health-related factors. [18, 19]. Job strain was measured at study baseline in 1986 and 9 165 individuals with data on job strain were eligible for our meta-analyses. The study was approved by the ethics committee of the Finnish Institute of Occupational Health.

*Whitehall II*

The Whitehall II study is a prospective cohort study set up to investigate socioeconomic determinants of health. At study baseline in 1985-1988, 10 308 civil service employees (6 895 men and 3 413 women) aged 35-55 and working at 20 civil service departments in London were invited to participate in the study [20]. Job strain was measured at study baseline and 10 285 men and women had data on job strain and were eligible for our meta-analyses. The Whitehall II study protocol was approved by the University College London Medical School committee on the ethics of human research. Written informed consent was obtained at each data collection wave.

*WOLF (Work, Lipids, and Fibrinogen) Stockholm and WOLF Norrland studies*

The WOLF (Work, Lipids, and Fibrinogen) Stockholm study is a prospective cohort study of 5 698 people (3 239 men and 2 459 women) aged 19–70 and working in companies in Stockholm county [21]. WOLF Norrland is a prospective cohort of 4 718 participants aged 19-65 working in companies in Jämtland and Västernorrland counties [22]. At study baseline the participants underwent a clinical examination and completed a set of health questionnaires. For WOLF Stockholm, the baseline assessment was undertaken at 20 occupational health units between November 1992 and June 1995 and for WOLF Norrland at 13 occupational health service units in 1996-98. The Regional Research Ethics Board in Stockholm, and the ethics committee at Karolinska Institutet, Stockholm, Sweden approved the study.

References

1. Pelfrene, E., et al., *Scale reliability and validity of the Karasek `Job Demand-Control-Support’ model in the Belstress study.* Work & Stress,, 2001. **15**(4): p. 297-313.

2. De Bacquer, D., et al., *Perceived job stress and incidence of coronary events: 3-year follow-up of the Belgian Job Stress Project cohort.* Am J Epidemiol, 2005. **161**(5): p. 434-41.

3. Kristensen, T.S., et al., *The Copenhagen Psychosocial Questionnaire--a tool for the assessment and improvement of the psychosocial work environment.* Scand J Work Environ Health, 2005. **31**(6): p. 438-49.

4. Burr, H., et al., *Trends in the Danish work environment in 1990–2000 and their associations with labor-force changes.* Scand J Work Environ Health, 2003. **29**(4): p. 270-279.

5. Feveile, H., et al., *Danish Work Environment Cohort Study 2005: From idea to sampling design.* Statistics in Transition, 2007. **8**(3): p. 441-458.

6. Kivimaki, M., et al., *Socioeconomic Position, Co-Occurrence of Behavior-Related Risk Factors, and Coronary Heart Disease: the Finnish Public Sector Study.* Am J Public Health, 2007. **97**(5): p. 874-879.

7. Goldberg, M., et al., *Cohort profile: the GAZEL Cohort Study.* Int J Epidemiol, 2007. **36**(1): p. 32-9.

8. Zins, M., A. Leclerc, and M. Goldberg, *The French GAZEL Cohort Study: 20 Years of Epidemiologi REsearch.* Advances in Life Course Research, 2009. **14**: p. 135-146.

9. Korkeila, K., et al., *Non-response and related factors in a nation-wide health survey.* Eur J Epidemiol, 2001. **17**(11): p. 991-9.

10. Schmermund, A., et al., *Assessment of clinically silent atherosclerotic disease and established and novel risk factors for predicting myocardial infarction and cardiac death in healthy middle-aged subjects: rationale and design of the Heinz Nixdorf RECALL Study. Risk Factors, Evaluation of Coronary Calcium and Lifestyle.* Am Heart J, 2002. **144**(2): p. 212-8.

11. Stang, A., et al., *Baseline recruitment and analyses of nonresponse of the Heinz Nixdorf Recall Study: identifiability of phone numbers as the major determinant of response.* Eur J Epidemiol, 2005. **20**(6): p. 489-96.

12. Nielsen, M., T. Kristensen, and L. Smith-Hansen, *The Intervention Project on Absence and Well-being (IPAW): design and results from the baseline of a 5-year study.* Work and Stress, 2002. **16**: p. 191-206.

13. Nielsen, M.L., et al., *Impact of the psychosocial work environment on registered absence from work: a two-year longitudinal study using the IPAW cohort.* Work & Stress 2004. **18**(4): p. 323-335.

14. de Groot, W. and R. Dekker, *The Dutch System of Official Social Surveys*, in *EuReporting Working Paper*. 2001, Mannheim Centre for European Social Research: Mannheim.

15. Borritz, M., et al., *Burnout among employees in human service work: design and baseline findings of the PUMA study.* Scand J Public Health, 2006. **34**(1): p. 49-58.

16. Magnusson Hanson, L.L., et al., *Demand, control and social climate as predictors of emotional exhaustion symptoms in working Swedish men and women.* Scand J Public Health, 2008. **36**(7): p. 737-43.

17. Hasson, D., et al., *Prevalence and characteristics of hearing problems in a working and non-working Swedish population.* J Epidemiol Community Health, 2010. **64**(5): p. 453-60.

18. Kalimo, R. and S. Toppinen, *Organizational well-being: ten years of research and development: in a forest industry corporation*, in *Preventing Stress, Improving Productivity: European Case Studies in the Workplace*, M. Kompier and C. Cooper, Editors. 1999, Routledge: London. p. 52-85.

19. Vaananen, A., et al., *Engagement in cultural activities and cause-specific mortality: prospective cohort study.* Prev Med, 2009. **49**(2-3): p. 142-7.

20. Marmot, M.G., et al., *Health inequalities among British civil servants: the Whitehall II study.* Lancet, 1991. **337**(8754): p. 1387-93.

21. Peter, R., et al., *High effort, low reward, and cardiovascular risk factors in employed Swedish men and women: baseline results from the WOLF Study.* J Epidemiol Community Health, 1998. **52**: p. 540-547

22. Alfredsson, L., et al., *Job strain and major risk factors for coronary heart disease among employed males and females in a Swedish study on work, lipids and fibrinogen.* Scand J Work Environ Health, 2002. **28**(4): p. 238-48.
