# Supplementary material for: Job Strain and Tobacco Smoking: An Individual-Participant Data Meta-Analysis of 166 130 Adults in 15 European Studies
Source: PLoS One. 2012 Jul 6;7(7):e35463. doi: 10.1371/journal.pone.0035463 (PMC3391192; doi:10.1371/journal.pone.0035463)
Supplement: Appendix S2 — MOOSE checklist. (DOC) [file pone.0035463.s008.doc]

**MOOSE Checklist**

**Article details:**

**Title:** Job Strain and Tobacco Smoking: An Individual-participant Data Meta-analysis of 166 130 Adults in 15 European Studies

**Short title:** Job Strain and Smoking: A Meta-analysis

**Authors:** IPD-Work Consortium

| **Criteria** | | **Brief description of how the criteria were handled in the meta-analysis** |
| --- | --- | --- |
| **Reporting of background should include** | |  |
| √ | Problem definition | Introduction, 1st paragraph. |
| √ | Hypothesis statement | Introduction, 2nd paragraph. |
| √ | Description of study outcomes | Methods, Ascertainment of tobacco smoking and work stress. |
| √ | Type of exposure or intervention used | Methods, Ascertainment of tobacco smoking and work stress. |
| √ | Type of study designs used | Methods, Studies and participants and Appendix S1. |
| √ | Study population | Methods, Studies and participants; Appendix S1; Figure S1. |
| **Reporting of search strategy should include** | |  |
| √ | Qualifications of searchers | Not applicable. This is a collaborative meta-analysis of individual-level data and thus not based on a search of published literature. |
| √ | Search strategy, including time period included in the synthesis and keywords | Not applicable: see above. |
| √ | Databases and registries searched | Not applicable: see above. |
| √ | Search software used, name and version, including special features | Not applicable: see above. |
| √ | Use of hand searching | Not applicable: see above. |
| √ | List of citations located and those excluded, including justifications | Not applicable: see above. |
| √ | Method of addressing articles published in languages other than English | Not applicable: see above. |
| √ | Method of handling abstracts and unpublished studies | Not applicable: see above. |
| √ | Description of any contact with authors | Contact with investigators described in Methods |
| **Reporting of methods should include** | |  |
| √ | Description of relevance or appropriateness of studies assembled for assessing the hypothesis to be tested | Introduction; and Methods, Studies and participants-section. |
| √ | Rationale for the selection and coding of data | Methods. Figure S1. |
| √ | Assessment of confounding | Methods, Covariates; and Statistical analyses |
| √ | Assessment of study quality, including blinding of quality assessors; stratification or regression on possible predictors of study results | Methods, Ascertainment of tobacco smoking and work stress. |
| √ | Assessment of heterogeneity | Methods: Statistical analyses; Results, throughout the section. |
| √ | Description of statistical methods in sufficient detail to be replicated | Methods: Statistical analyses. |
| √ | Provision of appropriate tables and graphics | Figure S1. Tables 1-4. Tables S1-S3. |
| **Reporting of results should include** | |  |
| √ | Graph summarizing individual study estimates and overall estimate | Figure 1 in the main manuscript and Figures S2 and S3. |
| √ | Table giving descriptive information for each study included | Table 1. |
| √ | Results of sensitivity testing | Results. Figure S3. |
| √ | Indication of statistical uncertainty of findings | 95% confidence intervals have been reported for all effect estimates throughout the manuscript and uncertainty discussed in the Discussion-section. |
| **Reporting of discussion should include** | |  |
| √ | Quantitative assessment of bias | This is a collaborative meta-analysis, so publication bias is not applicable. Possible bias in the reporting of exposures and outcomes in Discussion. |
| √ | Justification for exclusion | As this is a collaborative meta-analysis, all studies in which the investigators were willing to participate were included. |
| √ | Assessment of quality of included studies | Discussion. |
| **Reporting of conclusions should include** | |  |
| √ | Consideration of alternative explanations for observed results | Discussion. |
| √ | Generalization of the conclusions | Conclusions. |
| √ | Guidelines for future research | Discussion. |
| √ | Disclosure of funding source | Funding, Financial Disclosure and Competing interests statement. |

**MOOSE flow chart:** We have not included a flow chart of the literature search process because ours is a collaborative meta-analysis of individual-level data and thus not based on a search of published literature. A flow chart of the studies included in the analyses is provided in Figure S1.
